# Dickkopf-1 is an immune infiltration-related prognostic biomarker of head and neck squamous cell carcinoma

**DOI:** 10.18632/aging.205563

**Published:** 2024-02-19

**Authors:** Chaofen Zhao, Lina Liu, Qianyong He, Yuanyuan Li, Jianglong Feng, Yue Chen, Yuxin Li, Xinyu Xu, Shaoyuan Zhu, Yuanmei Ye, Yajing Wen, Zhirui Zeng, Ding’an Zhou, Feng Jin

**Affiliations:** 1 Department of Oncology, The Affiliated Hospital of Guizhou Medical University, Guiyang, P.R. China; 2 Department of Oncology, The Affiliated Cancer Hospital of Guizhou Medical University, Guiyang, P.R. China; 3 Department of Oncology, The School of Clinical Medicine, Guizhou Medical University, Guiyang, P.R. China; 4 Department of Pathology, Affiliated Hospital of Guizhou Medical University, Guiyang, P.R. China; 5 Department of Radiation Oncology, Nanfang Hospital, Southern Medical University, Guangzhou, P.R. China; 6 Department of Physiology, The School of Basic Medicine, Guizhou Medical University, Guiyang, P.R. China; 7 Clinical Research Center, Affiliated Hospital of Guizhou Medical University, Guiyang, Guizhou, P. R. China

**Keywords:** Dickkopf-1, head and neck squamous cell carcinoma, prognosis, immune cell infiltration, immunotherapy

## Abstract

Immunotherapy is currently one of the most viable therapies for head and neck squamous cell carcinoma (HNSCC), characterized by high immune cell infiltration. The Wnt-signaling inhibitor and immune activation mediator, Dickkopf-1 (DKK1), has a strong correlation with tumor growth, tumor microenvironment, and, consequently, disease prognosis. Nevertheless, it is still unclear how DKK1 expression, HNSCC prognosis, and tumor-infiltrating lymphocytes are related. To better understand these associations, we examined how DKK1 expression varies across different tumor and normal tissues. In our study, we investigated the association between DKK1 mRNA expression and clinical outcomes. Next, we assessed the link between DKK1 expression and tumor immune cell infiltration. Additionally, using immunohistochemistry, we evaluated the expression of DKK1 in 15 healthy head and neck tissue samples, and the expression of CD3, CD4, and DKK1 in 27 HNSCC samples. We also explored aberrant DKK1 expression during tumorigenesis. DKK1 expression was remarkably higher in HNSCC tissues than in healthy tissues, and was shown to be associated with tumor stage, grade, lymph node metastasis, histology, and a dismal clinical prognosis in HNSCC. DKK1 expression in HNSCC tissues was inversely correlated with CD3+ (P < 0.0001) and CD4+ (P < 0.0001) immune cell infiltration, while that in immune cells was inversely associated with HNSCC prognosis. These findings offer a bioinformatics perspective on the function of DKK1 in HNSCC immunotherapy and provide justification for clinical research on DKK1-targeted HNSCC treatments. DKK1 is a central target for improving the efficacy of HNSCC immunotherapy.

## INTRODUCTION

Head and neck squamous cell carcinoma (HNSCC) ranks sixth among all cancers in terms of global prevalence [1]. Clinicians diagnose more than 600,000 cases of HNSCC globally [2]. Approximately 95% of head and neck cancers are HNSCC, which develops in the mouth, hypopharynx, throat, or oropharynx [3]. The five-year survival rate for patients with HNSCC is less than 50%, despite the existence of intensive multimodal treatment techniques, such as surgical intervention, chemotherapy, radiation, targeted therapy, and immune therapy [4]. This is primarily because 80–90% of individuals with advanced HNSCC experience local recurrence or distant metastases [5]. Therefore, identifying novel therapeutic targets or possible biological markers, as well as a comprehensive investigation of the molecular processes underlying HNSCC progression, will be crucial for developing additional treatment modalities.

Recent research indicates a correlation between tumor-infiltrating immune cells (TIICs) and HNSCC prognosis [6]. Numerous studies have demonstrated that the number of invading immune cells in HNSCC represents an antitumor immune response and is indicative of overall survival (OS) [6]. Clinicians will have access to more accurate prognostic data regarding HNSCC if they have a better understanding of immune activities in the tumor microenvironment (TME). Previous studies have shown a link between TIIC and the onset and development of HNSCC [7]. In HNSCC, tumor-infiltrating lymphocytes (TIL), particularly T helper 1 (Th1) cells, promote interferon-mediated signaling and increase the levels of programmed death ligand 1 (PD-L1) in tumor tissues, thereby protecting them from a tumor-directed immune response [8]. Macrophage infiltration is critical for tumor immune escape, angiogenesis, proliferation, and metastasis [9]. Circulating CD4+CD25+FoxP3+ regulatory T (Treg) cells are linked with a dismal prognosis in HNSCC [10]. While previous studies have linked elevated Treg levels to favorable prognosis in HNSCC [11], these findings collectively suggest that TIICs may be a viable treatment target for improving clinical outcomes in individuals with HNSCC.

Dickkopf-1 (DKK1; a DKK family member) inhibits the Wnt-signaling pathway, which, in several tissues and many tumors, regulates a variety of biological and cellular functions, such as cellular proliferation, invasion, motility, apoptotic activity, and metastatic spread, via both β-catenin-dependent and independent pathways [12]. Recently, dysregulated DKK1 expression was identified as a possible biological marker of cancer progression and a prognostic factor for a variety of malignancies [13]. Recent research in colorectal cancer has shown that, due to mismatch repair defects, DKK1 can inactivate CD8+T lymphocytes, thereby inhibiting the tumor response to PD-1 blockade therapy [14]. Another study demonstrated that DKK1 overexpression influences the immune cell population with anti-tumor antibody effects within the TME by reducing CD45+leukocyte infiltration levels and decreasing the abundance of natural killer cells (NKs) and CD8+T cells [15]. In summary, DKK1 exerts its immunomodulatory effects by enhancing Th2 cell responses, impairing T cell function via myeloid-derived suppressor cell (MDSC) regulation, and inhibiting the growth of CD8+ T lymphocytes and NK cells, thereby enhancing inflammation and cancer immune escape [16].

Therefore, further studies on the role of DKK1 in cancer are warranted. While DKK1 plays several roles in the TME, the fundamental processes involving TILs and their impact on HNSCC progression, including the potential association between DKK1 and TILs, remain unclear. The main objective of this study was to explore the relationship between DKK1 expression, HNSCC prognosis, and TILs.

## RESULTS

### Different cancers express different levels of DKK1 mRNA

**Figure 1:**
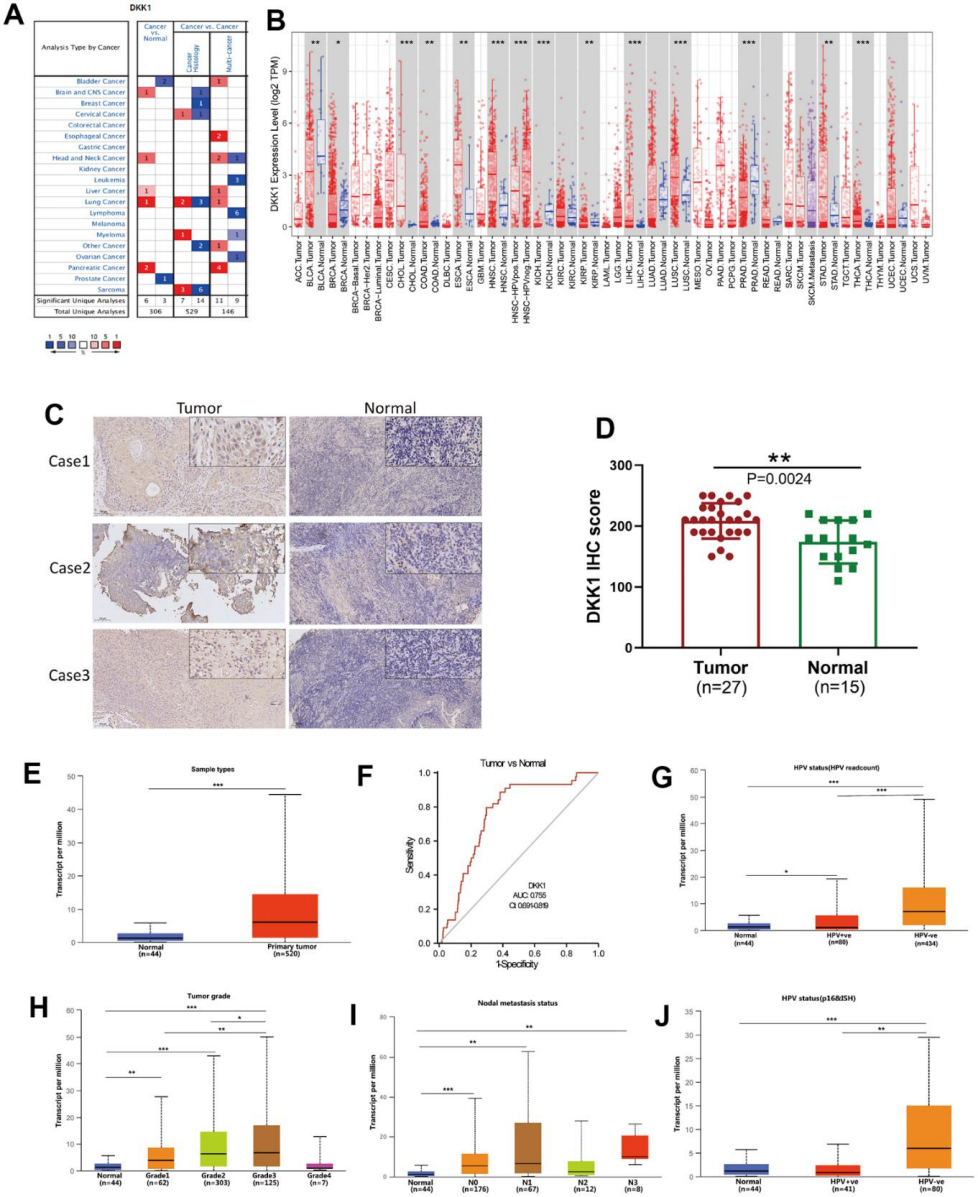
**DKK1 expression in different cancers and its relationship with individual clinical characteristics of HNSCC.** (**A**) DKK1 mRNA expression levels in different types of cancer tissues compared to that in normal tissues, based on information in the Oncomine database. (**B**) DKK1-expression levels in different cancers in the TIMER database. (**C**, **D**) DKK1-expression levels in representative HNSCC samples and normal tissues, as determined using IHC. The DKK1 levels were higher in HNSCC tissues than in normal tissues (P = 0.0024). (**E**) Differences in DKK1 expression in various HNSCC sample types, based on information deposited in the UALCAN database. (**F**) DKK1 exhibited a high diagnostic value for distinguishing between tumor and normal tissues. (**G**–**J**) DKK1 mRNA expression levels were remarkably correlated with the tumor grade (**H**), nodal-metastasis status (**I**), and HPV status (**G**, **J**) of patients with HNSCC, based on information from the UALCAN database (***p < 0.001, **p < 0.01, *p < 0.05).

The Oncomine database was used to analyze the mRNA-expression levels of DKK1 in distinct malignancies and matched normal tissues. DKK1 mRNA expression was higher in brain, central nervous system, head and neck, pancreatic, lung, esophageal, and liver cancers, but lower in bladder and prostate cancers (Figure 1A). To verify the DKK1 expression levels in various malignancies, we used the TIMER database to analyze mRNA sequencing (mRNA-Seq) data from The Cancer Genome Atlas (TCGA). Stomach adenocarcinoma (STAD), thyroid carcinoma (THCA), HNSCC, lung squamous cell carcinoma (LUSC), liver hepatocellular carcinoma (LIHC), esophageal carcinoma (ESCA), colon adenocarcinoma (COAD), and cholangiocarcinoma (CHOL) all showed considerably higher DKK1 expression. DKK1 levels, however, were substantially reduced in several cancers, including prostate adenocarcinoma (PRAD), kidney renal papillary cell carcinoma (KIRP), breast invasive carcinoma (BRCA), kidney chromophobe (KICH), and bladder urothelial carcinoma (BLCA). These findings show that DKK1 was expressed at varied levels in distinct malignancies, indicating that DKK1 performs a variety of functions in different tumors (Figure 1B).

### DKK1 expression was increased in HNSCC tissues and had a high diagnostic value

Next, we used immunohistochemistry (IHC) analysis to measure the level of DKK1 expression in 27 representative HNSCC samples and 15 normal head and neck tissues. The results indicated that HNSCC tissues had significantly higher DKK1 expression than normal tissues (P = 0.0024; Figure 1C, 1D). Data from the UALCAN, Oncomine, and TIMER databases were analyzed to verify this fact (Figure 1E). The results confirmed that an increase in DKK1 expression in patients with HNSCC can accurately distinguish between cancerous and non-cancerous tissues. Compared to normal tissues, the expression of DKK1 in patients was significantly upregulated, indicating that DKK1 may serve as an important biomarker for evaluating the condition of HNSCC (Figure 1F). Furthermore, DKK1 expression was linked to nodal metastatic status, tumor grade, individual cancer stage, and presence of HPV in HNSCC (Figure 1G–1J).

### Prognostic value of DKK1 mRNA expression in human cancers

**Figure 2:**
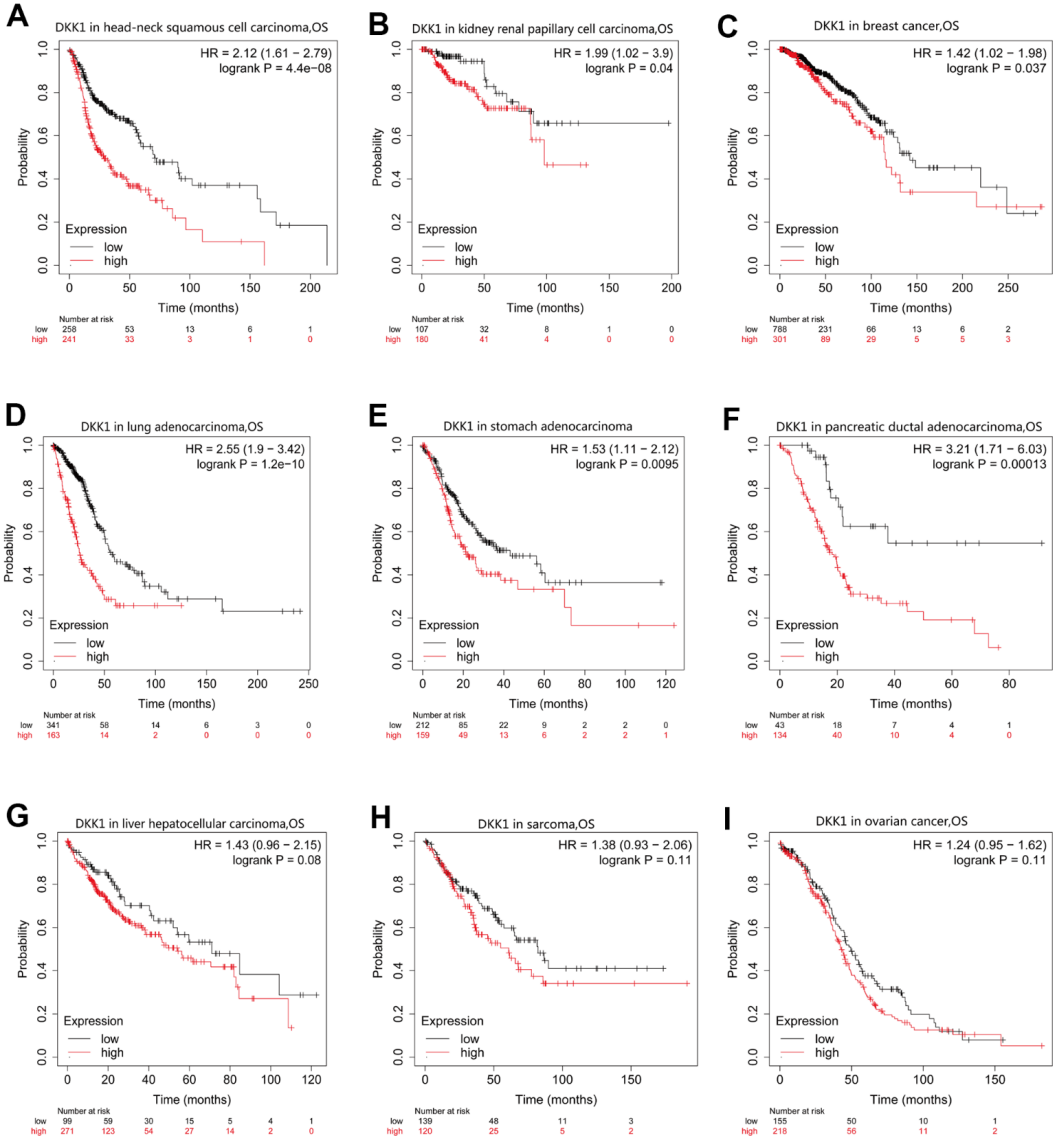
**OS curves for patients with nine different cancer types generated by analyzing mRNA-Seq data from TCGA with KM plotter databases.** (**A**–**G**) High DKK1 expression correlated with a poor OS in patients with HNSCC (n = 500), KIRP (n = 288), BRCA (n = 1090), LUAD (n = 513), STAD (n = 375), PAAD (n = 177), and LIHC (n = 371). (**H**, **I**) Survival differences among patients with SARC (n = 259) and OV (n = 374), with high and low DKK1 levels.

**Figure 3:**
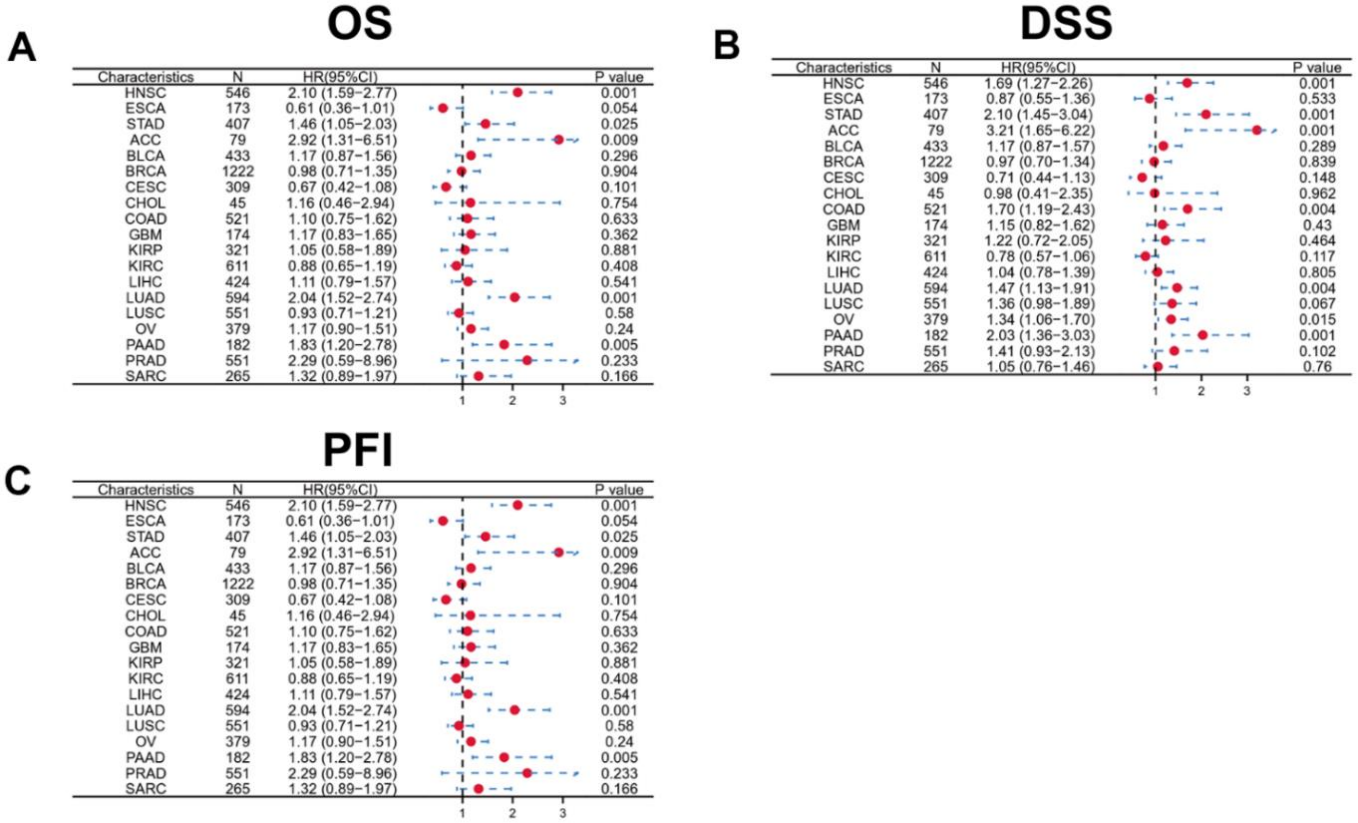
**Forest plot showing the prognostic significances for different cancer subgroups based on high or low DKK1 expression.** (**A**–**C**) Prognostic HRs related to DKK1 expression in various cancers in terms of OS (**A**), DSS (**B**), and PFI (**C**).

To determine the prognostic significance of DKK1 expression in human malignancies, we searched the Kaplan–Meier (KM) plotter database. Patients with HNSCC, LUAD, BRCA, KIRP, pancreatic ductal adenocarcinoma (PAAD), LIHC, and STAD who had high DKK1 levels had shorter survival times (Figure 2A–2G). In contrast, patients with sarcoma and ovarian cancer who expressed high levels of DKK1 showed little effect on their OS (Figure 2H, 2I). Analyzing OS values obtained using the KM Plotter, we applied the “survival” and “ggplot2” packages in R software to assess multiple clinical prognostic markers associated with DKK1 expression across various malignancies. In patients with HNSCC, STAD, ACC, LUAD, or PADD, a forest plot demonstrated that elevated DKK1 expression was a risk factor for unfavorable OS, progression-free interval (PFI), and disease-specific survival (DSS) (Figure 3A–3C). These results indicate that patients with HNSCC have poor prognosis when their DKK1 expression is upregulated.

### Association of DKK1 expression with clinical subtypes of HNSCC

We screened the KM Plotter database to assess the link between DKK1 levels and several clinicopathological factors associated with HNSCC (Table 1). A poor HNSCC prognosis was linked to DKK1 upregulation in grade 1 (OS: HR = 3.81, P = 0.0049), grade 2 (OS: HR = 2.02, P = 6.8e−05; RFS: HR = 3.36, P = 0.019), grade 3 (OS: HR = 2.69, P = 0.00027), stage 2 (OS: HR = 2.94, P = 0.0097), stage 3 (OS: HR = 5.52, p = 0.00022), stage 4 (OS: HR = 1.73, P = 0.0026), and both males (OS: HR = 2.3, P = 2.1e−06), and females (OS: hazard ratio [HR] = 2.49, P = 0.0033). Likewise, DKK1 upregulation was linked to lower levels of OS and RFS in patients with high mutation burdens (OS: HR = 2.48, P = 1.3e−06; RFS: HR = 0.25, p = 0.027) and low mutation burdens (OS: HR = 1.91, P = 0.0026). Based on these clinicopathological factors, DKK1 upregulation in patients with HNSCC may indicate a poor disease prognosis.

**Table 1:** Correlation of DKK1 mRNA expression and the prognosis in HNSCC with different clinical subtypes, based on the Kaplan–Meier plotter.

**Clinical subtypes**	**HNSCC**				
	**OS**			**RFS**	
	**HR**	**P**		**HR**	**P**
Gender					
Female (n = 4305)	2.49 (1.33-4.67)	0.0033		0.34 (0.11-1.02)	0.043
Male (n = 3184)	2.3 (1.61-3.27)	2.1e−06		2.76 (0.96-7.99)	0.051
Stage					
1 (n = 1790)	6.71 (0.69-65.63)	0.06		0.35 (0.08-1.59)	0.16
2 (n = 1707)	2.94 (1.25-6.93)	0.0097		0 (0-Inf)	0.11
3 (n = 1269)	5.52 (2.03-14.96)	0.00022		2.34 (0.6-9.08)	0.21
4 (n = 676)	1.73 (1.2-2.47)	0.0026		-	-
Grade					
1 (n = 304)	3.81 (1.41-10.29)	0.0049		2.36 (0.47-11.94)	0.29
2 (n = 1297)	2.02 (1.42-2.87)	6.8e−05		3.36 (1.15-9.87)	0.019
3 (n = 1510)	2.69 (1.54-4.67)	0.00027		0.3 (0.06-1.51)	0.12
4 (n = 93)	-	-		-	-
Mutation burden					
High (n = 3510)	2.48 (1.7-3.63)	1.3e−06		0.25 (0.07-0.94)	0.027
Low (n = 3390)	1.91 (1.25-2.94)	0.0026		2.3 (0.91-5.85)	0.071

### Correlation between immune infiltration and DKK1 expression in HNSCC

**Figure 4:**
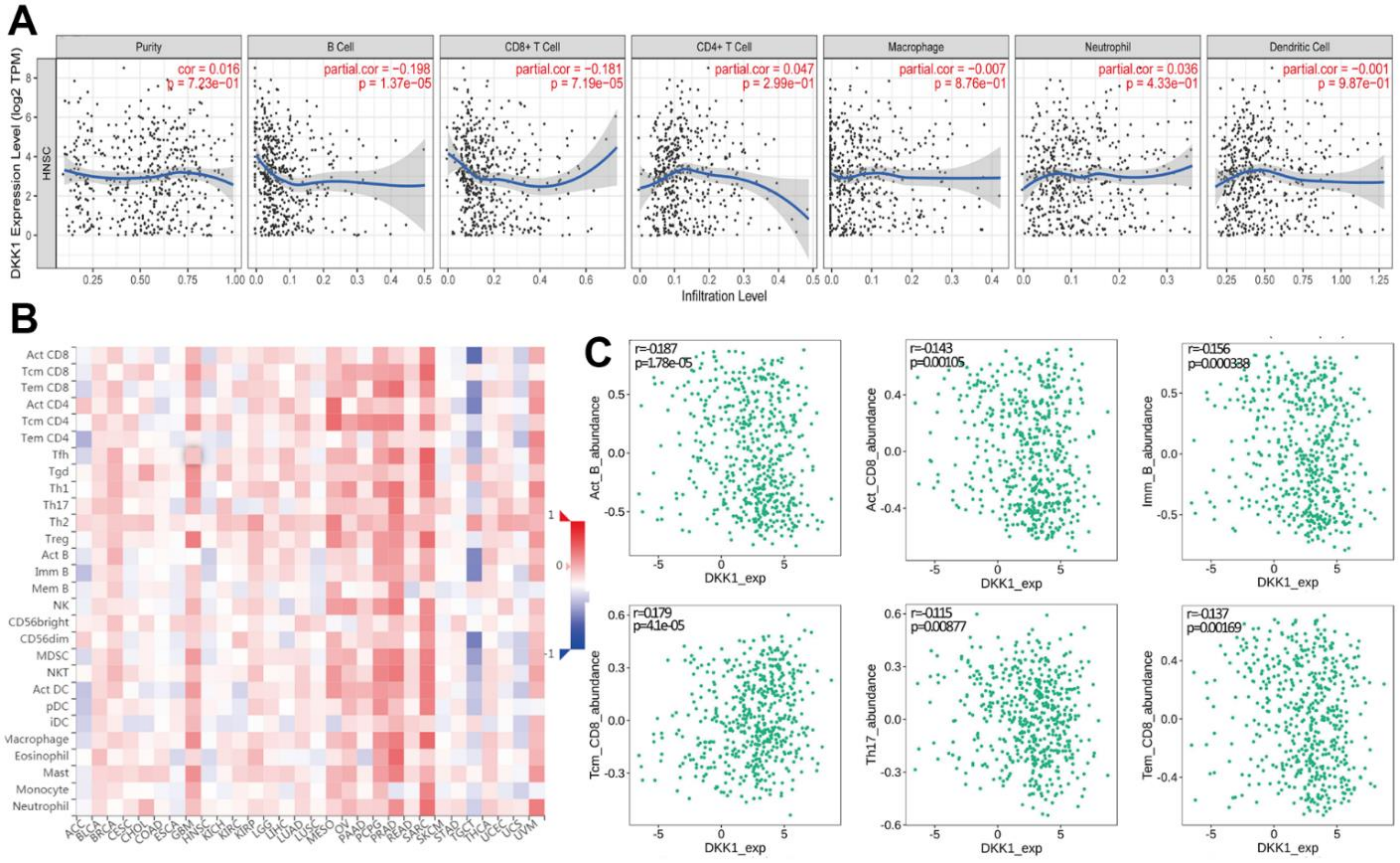
**Correlation between DKK1 expression and immune cell infiltration levels in human cancers, based on information in the TIMER and TISIDB databases.** (**A**) DKK1 expression levels in HNSCC tissues correlated negatively with the levels of B cell and CD8+ T cell infiltration into tumors. (**B**) Relations between DKK1 expression and the levels of 28 types of TILs in human heterogeneous cancers. (**C**) The top six TILs displayed significant differences in Spearman correlation coefficients with DKK1-expression differences in HNSCC.

Although DKK1 has been linked to immune infiltration and prognosis in LIHC, ESCA, and PAAD, how DKK1 immune infiltration is related to HNSCC remains unknown. The link between DKK1 expression and the infiltrating levels of immune cells in distinct cancer subtypes was examined using the TIMER database. The findings indicated a substantial inverse correlation between DKK1 expression, CD8+ T cell (r = -0.181, p = 7.19e−05), and B cell (r = -0.198, p = 1.37e−05) infiltration in HNSCC (Figure 4A). Additionally, we used a Cox proportional hazards model with TIMER to assess the prognostic significance of DKK1 levels and TIICs in HNSCC. The findings showed a significant correlation between DKK1 expression and the clinical prognosis of HNSCC (p< 0.001). (Table 2). Next, we probed the TISIDB database to examine the link between DKK1 levels and the 28 TIIC subtypes. Based on these findings, DKK1 was linked to 11 immune cell subtypes in HNSCC (Figure 4B and Table 3). We discovered that DKK1 expression was moderately linked to immature B cells (r = -0.156, p = 0.000338), activated B cells (r = -0.187, p = 1.78e−05), Th17 cells (r = -0.115, p = 0.00877), effector memory CD8+ T cells (r = -0.137, p = 0.00169), central memory CD8+ T cells (r = 0.179, p = 4.1e−05), and activated CD8+ T cells (r = -0.143, p = 0.00105) (Figure 4C). These results suggest that DKK1 is involved in the regulation of TIICs in HNSCC.

**Table 2:** Cox proportional hazard model of DKK1 and six tumor-infiltrating immune cell types in HNSCC, as determined using the TIMER database.

	**HNSCC**				
	**coef**	**HR**	**95% CI_l**	**95% CI_u**	**p-value**
B cells	-1.769	0.170	0.013	2.155	0.172
CD8+ T cells	-0.892	0.410	0.064	2.638	0.348
CD4+ T cells	-2.990	0.050	0.002	1.136	0.060
Macrophages	2.269	9.668	0.800	116.786	0.074
Neutrophils	-0.976	0.377	0.021	6.831	0.509
Dendritic cells	0.863	2.371	0.534	10.520	0.256
DKK1	0.133	1.142	1.060	1.230	0.000

**Table 3:** Correlations between DKK1-expression levels and tumor lymphocyte infiltration in HNSCC, as determined using the TISIDB database.

	**HNSCC**	
	**r**	**p**
Activated CD8+ T cell (Act _CD8)	-0.143	0.00105
Central memory CD8+ T cell (Tcm _CD8)	0.179	4.1e−05
Effector memory CD8+ T cell (Tem _CD8)	-0.137	0.00169
Activated CD4 T+ cell (Act _CD4)	-0.011	0.795
Central memory CD4+ T cell (Tcm _CD4)	0.1	0.0227
Effector memory CD4+ T cell (Tem _CD4)	-0.078	0.0751
T follicular helper cell (Tfh)	-0.073	0.0956
Gamma delta T cell (Tgd)	0.056	0.204
Type-1 T helper cell (Th1)	-0.074	0.0908
Type-17 T helper cell (Th17)	-0.115	0.00877
Type-2 T helper cell (Th2)	0.067	0.126
Regulatory T cell (Treg)	0.019	0.671
Activated B cell (Act_B)	-0.187	1.78e−05
Immature B cell (Imm_B)	-0.156	0.000338
Memory B cell (Mem_B)	-0.026	0.561
Natural killer cell (NK)	-0.066	0.129
CD56bright natural killer cell (CD56bright)	0.104	0.017
CD56dim natural killer cell (CD56dim)	-0.068	0.119
Myeloid-derived suppressor cell (MDSC)	-0.1	0.0225
Natural killer T cell (NKT)	0.038	0.386
Activated dendritic cell (Act_DC)	-0.023	0.594
Plasmacytoid dendritic cell (pDC)	0.045	0.306
Immature dendritic cell (iDC)	-0.044	0.314
Macrophage (Macrophage)	-0.011	0.807
Eosinophil (Eosinophil)	-0.144	0.001
Mast (Mast)	-0.099	0.0235
Monocyte (Monocyte)	0.005	0.914
Neutrophil (Neutrophil)	0.052	0.239

### Correlation between immune cell markers and DKK1 expression

**Figure 5:**
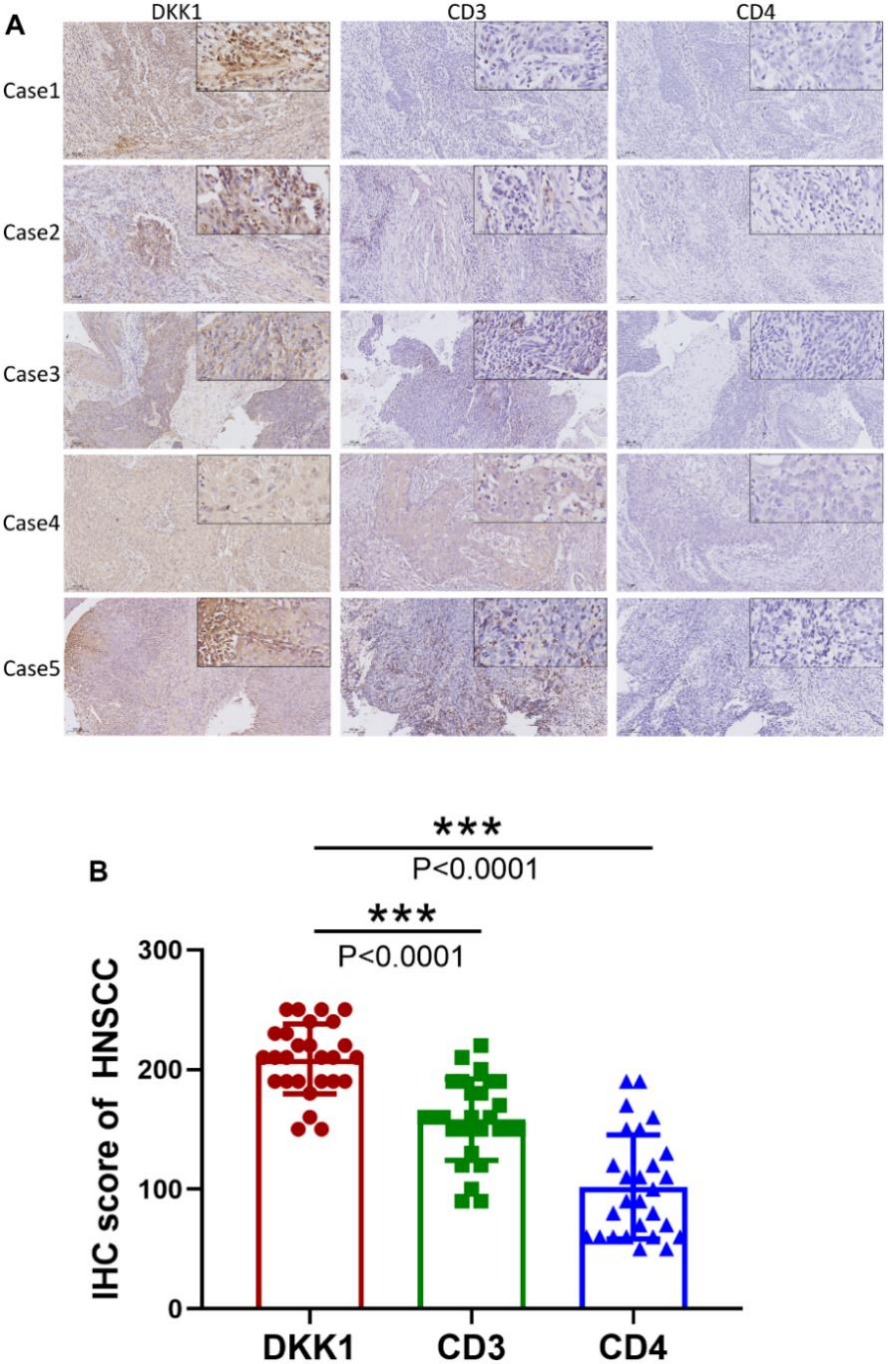
**Increased DKK1 expression and decreased CD3 and CD4 expression in HNSCC.** (**A**) IHC of DKK1, CD3, and CD4 expression in representative HNSCC samples. (**B**) Scatter plots showing that the DKK1, CD3, and CD4 IHC scores differed significantly in HNSCC (***p < 0.001).

Using the TIMER database, we analyzed the link between the abundance of DKK1 and TIIC gene markers in HNSCC tissues. Our findings demonstrated an inverse link between the levels of DKK1 in HNSCC tissues and the B cell and CD8+ T lymphocyte marker genes. A striking observation was that DKK1 expression in HNSCC was linked to markers of various B cell subtypes (CD79A and CD19), Tregs (STAT5B, FOXP3, and TGF-β), Th2 cells (GATA3, STAT6, and STAT5A), Th1 cells (TBX21, STAT4, and IFNG), tumor-associated macrophages (TAMs) (CCL2 and CD68), neutrophils (CCR7 and ITGAM), NK cells (KIR3DL1), monocytes (CD86), M1 macrophages (PTGS2 and IRF5), M2 macrophages (VSIG4 and CD163), dendritic cells (NRP1 and CD1C), T cell-exhaustion (PDCD1), general T cells (CD2, CD3E, and CD3D), and CD8+ T lymphocytes (CD8A and CD8B) (Table 4). Additionally, IHC analysis revealed that DKK1 expression was upregulated, whereas CD3 and CD4 expression was downregulated in HNSCC tissues (P < 0.0001) (Figure 5A, 5B). Furthermore, DKK1 expression was inversely linked to CD3+ and CD4+ immune cell infiltration in HNSCC. In summary, DKK1 was implicated in the regulation of TIICs in HNSCC.

**Table 4:** Correlation analysis between DKK1, related genes, and markers of immune cells, as determined using the TIMER database.

**Description**	**Gene marker**	**HNSCC**			
		**None**		**Purity**	
		**cor**	**p**	**cor**	**p**
B cell	CD19	-0.162	1.95e−04	-0.171	1.42e−04
	CD79A	-0.15	5.88e−04	-0.155	5.77e−04
CD8+ T cell	CD8A	-0.146	8.15e−04	-0.138	2.09e−03
	CD8B	-0.181	3.06e−05	-0.175	9.13e−05
T cell (general)	CD3D	-0.179	4.05e−05	-0.172	1.26e−04
	CD3E	-0.148	6.94e−04	-0.139	2.08e−03
	CD2	-0.153	4.53e−04	-0.145	1.29e−03
T cell exhaustion	CTLA4	0.065	1.35e−01	-0.06	1.85e−01
	LAG3	-0.08	6.69e−02	-0.071	1.15e−01
	HAVCR2	0.001	8.7e−01	0.031	4.98e−01
	GZMB	-0.082	6.01e−02	-0.074	1.03e−01
	PDCD1	-0.117	7.49e−03	-0.109	1.52e−02
Dendritic cell	ITGAX	-0.01	8.19e−01	0.004	9.30e−01
	NRP1	0.185	2.15e−05	0.208	3.38e−06
	CD1C	-0.126	4.04e−03	-0.108	1.64e−02
	HLA-DPA1	-0.07	1.09e−01	-0.051	2.58e−01
	HLA-DRA	-0.07	1.13e−01	-0.054	2.30e−01
	HLA-DQB1	-0.044	3.21e−01	-0.031	4.95e−01
	HLA-DPB1	-0.107	1.46e−02	-0.095	3.48e−02
M1 Macrophage	PTGS2	0.124	4.66e−03	0.123	6.27e−03
	IRF5	-0.128	3.37e−03	-0.124	6.07e−03
	NOS2	-0.005	9.17e−01	0.003	9.44e−01
M2 Macrophage	MS4A4A	0.07	1.08e−01	0.095	3.49e−02
	VSIG4	0.098	2.56e−02	0.123	6.27e−03
	CD163	0.076	8.43e−02	0.098	3.01e−02
Monocyte	CSF1R	-0.015	7.35e−01	0.006	8.89e−01
	CD86	0.115	8.52e−03	0.145	1.28e−03
Natural killer cell	KIR2DS4	-0.078	7.67e−02	-0.075	9.77e−02
	KIR3DL3	-0.051	2.47e−01	-0.034	4.56e−01
	KIR3DL2	-0.054	2.18e−01	-0.033	4.67e−01
	KIR3DL1	-0.096	2.84e−02	-0.105	1.95e−02
	KIR2DL4	-0.032	4.59e−01	-0.023	6.17e−01
	KIR2DL3	-0.066	1.31e−01	-0.052	2.52e−01
	KIR2DL1	0.028	5.22e−01	0.042	3.49e−01
Neutrophil	CCR7	-0.138	1.59e−03	-0.137	2.33e−03
	ITGAM	0.1	2.22e−02	-0.08	7.48e−02
	CEACAM8	−0.016	7.14e−01	-0.022	6.30e−01
TAM	CCL2	0.075	8.8e−02	0.094	3.76e−02
	IL10	-0.005	9.06e−01	-0.012	7.88e−01
	CD68	0.11	1.16e−02	0.135	2.77e−03
Tfh	BCL6	-0.037	4e−01	-0.035	4.35e−01
	IL21	-0.064	1.44e−01	-0.042	3.51e−01
Th1	TBX21	-0.12	6.22e−03	-0.11	1.42e−02
	STAT4	0.098	2.54e−02	0.126	5.23e−03
	STAT1	0.047	2.82e−01	0.065	1.51e−01
	IFNG	-0.11	1.19e−02	-0.101	2.44e−02
	IL13	-0.067	1.25e−01	-0.049	2.82e−01
Th2	GATA3	-0.073	9.56e−02	-0.076	9.17e−02
	STAT6	0.065	1.39e−01	0.093	4.01e−02
	STAT5A	-0.103	1.86e−02	0.095	2.49e−02
Th17	STAT3	-0.063	1.49e−01	-0.042	3.52e−01
	IL17A	-0.095	3.03e−02	-0.081	7.30e−02
Treg	FOXP3	-0.098	2.54e−02	-0.088	5.16e−02
	CCR8	-0.028	5.28e−01	-0.008	8.52e−01
	STAT5B	0.108	1.37e−02	0.121	7.07e−03
	TGFB1	0.199	4.63e−06	0.209	1.84e−06

### Prognostic value of DKK1 expression in HNSCC based on immune cells

**Figure 6:**
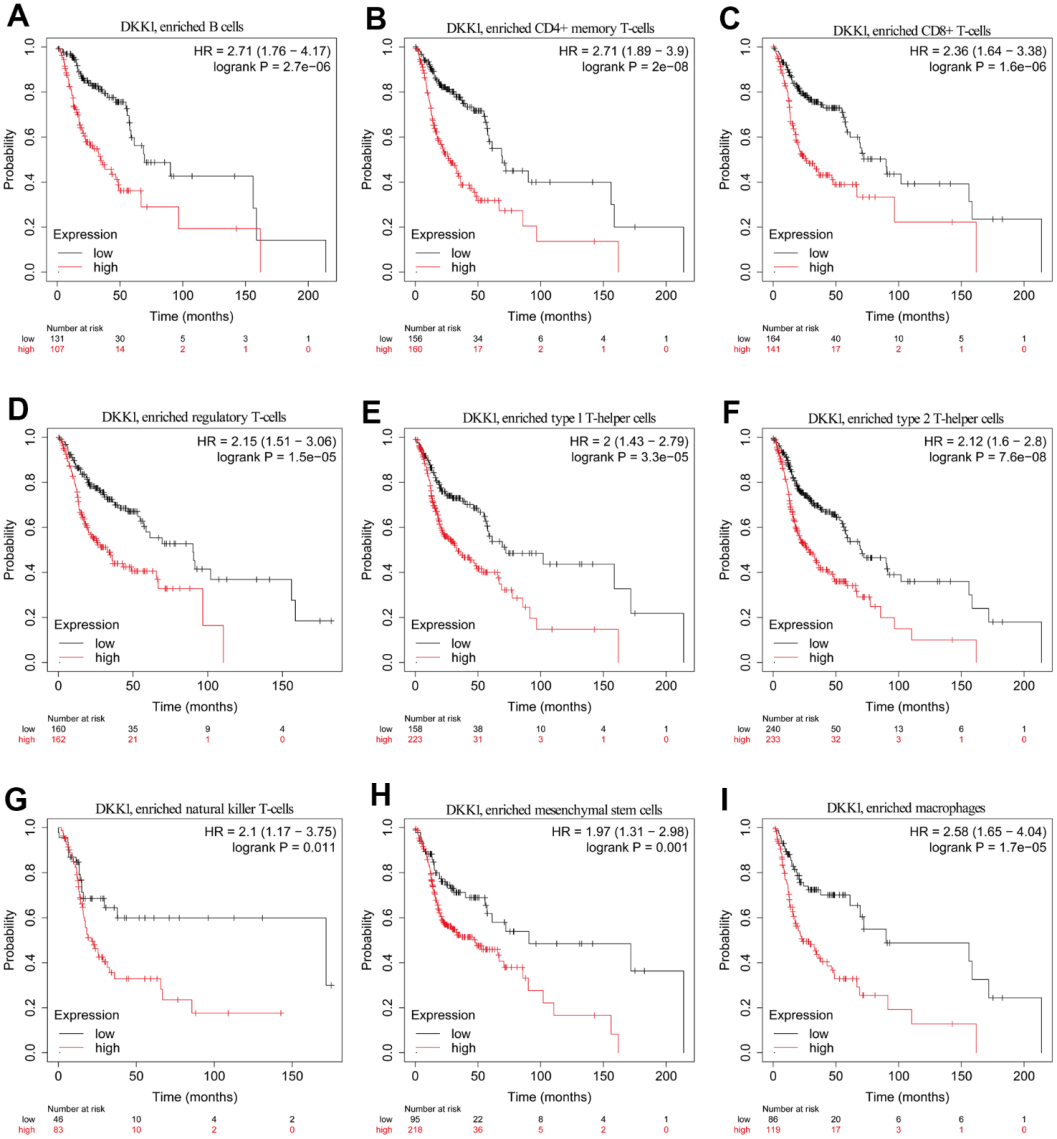
**Comparison of KM survival curves of patients with low and high DKK1 expression in HNSCC, based on their immune subtypes.** (**A**–**I**) Patients with HNSCC and high DKK1 levels in B cells, CD4+ memory T cells, CD8+ T cells, Treg cells, Th1 cells, Th2 cells, NK T cells, mesenchymal stem cells, and macrophages had a worse OS.

The findings of this study prove that immune cell infiltration in HNSCC is linked to the degree of DKK1 expression. Patients with HNSCC had poor prognosis when DKK1 was upregulated. Thus, we postulate that DKK1 might, in part, influence the prognosis of patients with HNSCC via immune infiltration. With the aid of a KM plotter, we discovered that patients with HNSCC had a dismal prognosis when DKK1 expression was higher in macrophages (p = 1.7e−05), mesenchymal stem cells (p = 0.001), NK T cells (p = 0.011), Th1 cells (p = 3.3e−05), Th2 cells (p = 7.6e−08), Treg cells (p = 1.5e−05), CD8+ T cells (1.6e−06), CD4+ memory T cells (p = 2e−08), and B cells (p = 2.7e−06) (Figure 6A–6I). According to this data, patients with HNSCC and high DKK1 levels may have prognostic effects from immune infiltration.

### Gene expression, CNV, and mutation-feature analysis of DKK1 in HNSCC

**Figure 7:**
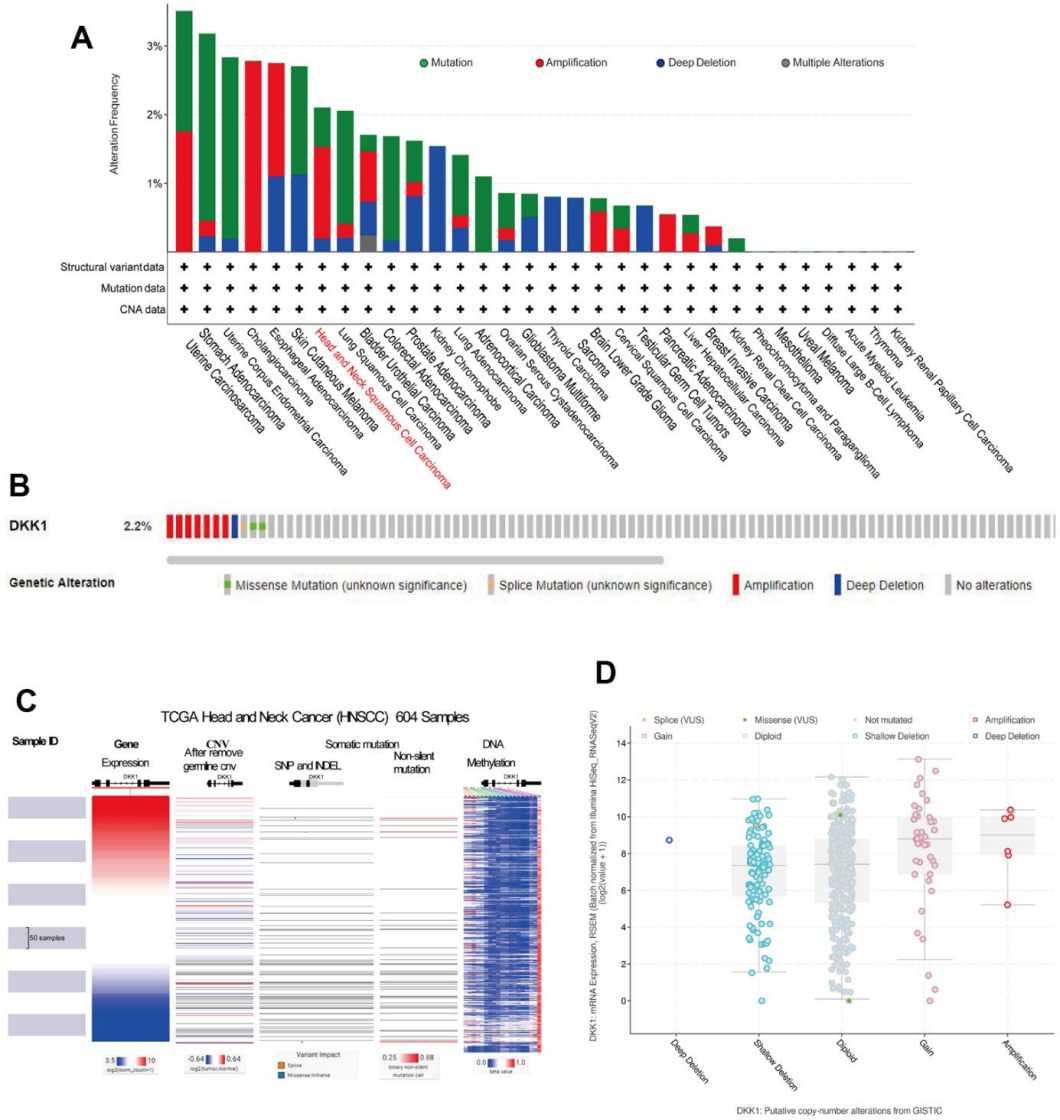
**Gene expression, CNV, and mutation feature analysis of DKK1 expression.** (**A**) CNA and mutation-frequency data related to DKK1 expression in different tumor types were accessed from cBioPortal. (**B**) The alteration frequencies of different mutation types in HNSCC were displayed using the cBioPortal tool. (**C**) Heatmap showed correlations between DKK1 mRNA expression levels and CNVs, somatic mutations, and DNA methylation in HNSCC, based on information in the UCSC Xena database. (**D**) A plot showed the relationship between DKK1 mRNA expression and CNAs in the DKK1 gene in HNSCC tumors, as determined using the cBioPortal tool.

DKK1 was expressed at substantially higher levels in HNSCC samples. Consequently, we investigated the cause of the upregulated DKK1 levels. Notably, tumor onset and progression were influenced by DNA methylation, gene mutations, and copy-number variations (CNVs). We used the web-based cBioPortal tool and TCGA cohort data to examine DKK1 genetic alterations, which primarily encompassed mutations, amplifications, and copy number deletions, in patients with HNSCC. Copy number alterations (CNAs) of the amplification type, with an alteration frequency of approximately 2.2%, were the primary type in patients with HNSCC (Figure 7A, 7B). Figure 7B provides more information on the various genetic alterations in DKK1. Using the UCSC Xena database, we verified the levels of CNV, somatic mutations, and DNA methylation of DKK1 in HNSCC. DKK1 mRNA expression in HNSCC was associated with CNVs and DNA methylation; however, it was not associated with somatic mutations, as illustrated in the heatmap (Figure 7C). According to cBioPortal data, samples with amplified DKK1 had higher levels of DKK1 mRNA (Figure 7D). Thus, our findings suggest that DNA methylation and CNV play a role in DKK1 overexpression in HNSCC.

## DISCUSSION

In this study, we examined DKK1 expression and its link to HNSCC prognosis using the UALCAN, TIMER, and Oncomine datasets, as well as the KM Plotter website. In addition, we examined the relationship between DKK1 and TIICs in the TME using the TIMER portal and TISIDB database. Through IHC, we probed the expression of DKK1, CD3, and CD4 in HNSCC tissues, and DKK1 in normal head and neck tissues. Moreover, using the cBioPortal and UCSC Xena databases, we probed the molecular mechanisms of DKK1 dysregulation, with a focus on CNVs, somatic cell mutations, and DNA methylation. Our results highlight the crucial role of DKK1 in HNSCC prognostication and elucidate the underlying mechanism by which DKK1 expression may regulate TIICs.

DKK1 is a 35-kDa protein belonging to the DKK gene family, and is known for its involvement in the regulation of tumor proliferation, invasion, and metastasis, as well as the extracellular microenvironment [16, 17]. Herein, we examined DKK1 expression profiles in HNSCC for the first time, together with their prognostic significance and associations with TIICs, CNVs, DNA methylation, and somatic mutations.

We discovered that, compared to healthy samples, DKK1 expression was markedly upregulated in HNSCC (Figure 1A–1E). Furthermore, TCGA data for HNSCC tissues showed that DKK1 expression had a high diagnostic value (Figure 1F). In addition, DKK1 mRNA expression levels were significantly related to the cancer stage, HPV status, nodal-metastasis status, and tumor grade of individuals with HNSCC. Elevated DKK1 levels were also linked to HPV-negative status, a high mutation load, lymph node metastasis, and late-stage tumors with high tumor grades (Figure 1G–1J and Table 1). Compared with HPV-negative HNSCC, DKK1 mRNA levels were reduced in HPV-positive HNSCC (Figure 1J), indicating that DKK1 is specifically involved in HPV-negative HNSCC and plays a crucial role in the progression and metastasis of HNSCC. These findings are consistent with those of previous studies. For instance, it has been demonstrated that DKK1 is elevated in specific tumor tissues and is linked to clinicopathological variables, such as high tumor grade, lymph node metastasis, late N stage, and tumor-node-metastasis staging [18–20].

According to the survival and forest plot analyses, DKK1 upregulation was linked to unfavorable OS, DSS, and PFI rates in patients with HNSCC. Several preclinical studies on both *in vitro* cellular and *in vivo* animal models have demonstrated that DKK1 overexpression could accelerate tumor development, invasion, and metastatic spread [21, 22]; these inferences are in line with the findings of this investigation. According to Shi et al., patients with intrahepatic cholangiocarcinoma and DKK1 upregulation have dismal prognoses following surgery and lymphatic metastases [23]. Importantly, our findings suggest that DKK1 is a useful biological marker for HNSCC.

According to previous studies, DKK1 modulates immune cell function and serves as an immunosuppressor in the TME [16]. Based on these earlier findings, we examined whether DKK1 is linked to immune escape in HNSCC. In HNSCC, we discovered that CD8+ T cells, CD3+ T cells, CD4+ T cells, B cells, and Th17 cells were all related to the degree of DKK1 expression (Tables 2, 3 and Figures 4, 5). These findings indicate that DKK1 plays a role in TIICs regulation. According to Sui et al., DKK1 inhibits colorectal cancer tumor response to PD-1 blockade by inactivating CD8+ T cells [14]. Another study demonstrated that tumors of metastatic castration-resistant prostate cancer with high DKK1 expression exhibited a CD8+ T cell-poor TME infiltrated by immature M0 and M2 macrophages [24]. The findings of these earlier investigations are supported by our present analysis.

Additionally, we examined HNSCC immunotype biomarkers in more detail. We discovered that DKK1 was negatively associated with various immune cell markers in HNSCC, after accounting for cell purity (Table 4). Our analysis also illustrates that immune cell infiltration and DKK1 expression are associated with HNSCC. DKK1 expression in HNSCC was inversely linked to CD3 and CD4 expression (Figure 5), and DKK1 may exert negative modulatory effects on CD8+, B, and CD4+ memory T cells, leading to T cell exhaustion. Notably, Treg and T-cell exhaustion indicators, such as PDCD1 and FOXP3, were inversely linked to DKK1 upregulation. In the cancer microenvironment, FOXP3 is a viable target for detecting Tregs, which substantially promotes tumor immune evasion [25]. In HNSCC samples, significant associations were found between DKK1 expression and a range of Th subtypes (Th1, Th2, and Th17). These links represent the subtle ways in which DKK1 potentially controls T cell activity in HNSCC. Moreover, the infiltration levels of most MDSCs, including macrophages and mesenchymal stem cells, in HNSCC were adversely associated with DKK1 expression. Predicated on these findings, we hypothesized that DKK1 influences immune cell recruitment and regulation, which may contribute to poor prognosis in HNSCC. Overall, our findings imply that DKK-1 may indirectly stimulate tumor development by inducing immunosuppressive effects in malignant tumors.

Based on an examination of the KM plotter database and data from HNSCC cohorts, we discovered that elevated DKK1 expression in different types of immune cells was associated with a dismal prognosis. Tregs aid in the immunological escape of tumors by blocking antitumor T-effector responses [26]. Dendritic cells aid in the metastatic spread of tumors by lowering the levels of cytotoxic CD8+ T cells and increasing the number of Tregs [27]. In the past, MDSCs were referred to as a heterogeneous group of immature myeloid cells recruited by tumors to promote CD8+ T cell tolerance [28]. According to newly discovered experimental data, the accumulation of MDSCs by DKK1 has been shown to contribute to T cell differentiation and the induction of cancer immune surveillance evasion [16]. These insights help to clarify how immune infiltration influences the prognosis of patients with HNSCC and DKK1 overexpression.

The regulation of immunological tolerance and cancer progression through genetic and epigenetic alterations is a promising research field [29]. For instance, earlier research revealed that some PD-L1 mutants have structural differences that might result in abnormal PD-L1 production and immunosuppression [30, 31]. These findings indicate that epigenetic mechanisms play a critical role in regulating immune cell activity and promoting antitumor immunity. The various regulatory mechanisms linked to DKK1 expression represent diverse cellular localization functions in various cell types. According to preliminary research, CNAs and DNA methylation both genetically and epigenetically control DKK1 expression. Gene amplification, deletion, gain, diploidy, and other processes can result in DNA CNVs. CNVs play critical roles in pathogenesis and tumorigenesis. Zhang et al. illustrated that ablation of MT1 gene might function as a standalone prognostic indicator for hepatocellular cancer [32]. David et al. determined that DNA methylation is likely the mechanism controlling DKK1 expression in metastatic castration-resistant prostate cancer [24]. Another implication of these findings is that the suppression of therapeutic DNA methyltransferases, which is currently being investigated in clinical trials, may lead to resistance if DKK1 is upregulated. Therefore, we speculated that DNA hypomethylation and CNVs may be the causes of DKK1 upregulation in HNSCC. We plan to investigate this in future studies.

In hepatocellular carcinoma, inhibition of DKK1 enhances the anti-tumor efficacy of sorafenib via inhibition of the PI3K/Akt and Wnt/β-catenin pathways [33]. DKK1 promotes PD-L1 expression through the activation of Akt/β-catenin signaling, providing a potential strategy to enhance the clinical efficacy of PD-1/PD-L1 blockade therapy in patients [34]. In prostate cancer, the pro-cancer effect of DKK-1 is related to increased bone metastasis growth and decreased bone induction [35]. In HNSCC, the expression level of DKK1 was increased in patients with higher T-stage HNSCC, and this increase was related to shorter OS and PFS/DFS [36]. Our results suggest that DKK1 overexpression in HNSCC is strongly correlated with a range of clinical features, dismal prognosis, and immune filtration. DNA methylation and CNVs may be involved in DKK1 upregulation. Our study also suggests the intriguing potential of DKK1 in influencing the prognosis of patients with HNSCC by infiltrating the tumor immune system. Our findings thus pave the way for additional investigations into cancer immunotherapy for HNSCC. This is a pilot version of a larger study that includes validation using a prospectively registered study population. To confirm our findings, further trials will be conducted in the future.

However, this study has certain limitations. The clinical sample size was relatively small, and the mechanism underlying the relationship between DKK1 expression and immune cell infiltration was not thoroughly studied. We intend to explore the relationship between DKK1 expression and immune cell infiltration in subsequent research, and elucidate its specific mechanism of action to compensate for these limitations and deepen our understanding of DKK1 function. In summary, our findings offer a theoretical framework for the clinical investigation of HNSCC treatment through DKK1 gene targeting. DKK1 may play a noteworthy role in revolutionizing HNSCC immunotherapy and enhancing its therapeutic effects.

## MATERIALS AND METHODS

### Oncomine database

Oncomine is an online data-mining tool that includes a microarray database covering most human malignancies (https://www.oncomine.org). It is primarily employed to analyze gene expression levels, co-expression, enrichment, and interaction networks [37]. We examined DKK1 expression in several cancer types using the Oncomine database.

### TIMER database

The TIMER database (https://cistrome.shinyapps.io/timer/) is an online portal that facilitates thorough investigation of the expression of genes and TIICs in various types of cancers. This platform can be used to determine the abundance of immune cells infiltrating tumors (macrophages, B cells, dendritic cells, neutrophils, CD4+ T cells, and CD8+ T cells) [38]. Investigating the level of gene expression in healthy and cancerous tissues is another feature of the TIMER site. We investigated DKK1 expression in several tumors and healthy tissues from various malignancies using the TIMER platform. Additionally, we assessed the link between DKK1 expression in six distinct immune cell types that infiltrate tumors and biomarkers from 16 different immune cell types. Furthermore, we examined how the gene expression levels influencing the clinical prognosis of HNSCC are related to infiltrating immune cells (gene expression levels are presented as the log_2_ of the RSEM value).

### UALCAN database

Online studies of distinct gene expression levels in tumor and normal tissues are possible using the UALCAN database (http://ualcan.path.uab.edu/index.html). For the analysis of 31 different cancers, UALCAN utilizes clinical information and TCGA transcriptome sequencing data [39]. Additionally, UALCAN provides survival-prognosis information for 31 different tumor types based on variations in gene expression. Findings from the Oncomine database were verified using the UALCAN database, and correlations between DKK1 expression levels and clinicopathological variables were ascertained.

### Sample selection and data collection

We assessed DKK1 expression in 15 healthy head and neck tissue samples along with CD3, CD4, and DKK1 expression in 27 HNSCC samples. Samples from normal tissues (n = 15) and patients with nasopharyngeal carcinoma (n = 10), tongue carcinoma (n = 7), laryngeal carcinoma (n = 7), and other forms of head and neck carcinoma besides HNSCC (n = 3), were collected from December 2017 to August 2018 at the Affiliated Cancer Hospital of Guizhou Medical University and Affiliated Hospital of Guizhou Medical University. A competent pathologist assessed the IHC-labelled sections, and patients who met the criteria for an appropriate diagnosis were chosen for the study.

### Validation of diagnostic significance of genes

Using TCGA HNSCC data (https://portal.gdc.cancer.gov/), receiver-operating characteristic (ROC) curve analysis was conducted to examine the diagnostic performance of several genes. R (version 3.6.3) was used to analyze the ROC curve that predicted no TCGA HNSCC data [40]. Genes were deemed to have a strong diagnostic value if they had an area under the curve (AUC) >0.7.

### KM plotter

Prognostic analysis was performed using the KM plotter (http://kmplot.com/analysis/) [41]. The DKK1-expression levels in several tumor types were correlated with clinical outcomes using the KM plotter database. Based on the DKK1 expression levels in the corresponding immune cell subgroups, we performed prognostic analysis using the KM website. We calculated the HRs for the log-rank p-values and 95% confidence intervals (CIs).

### TISIDB database

TISIDB is an integrative archive platform for studying interactions between tumors and the immune system (http://cis.hku.hk/TISIDB/) [42]. Using data from the TISIDB database, we analyzed Spearman correlations between DKK1 levels and TILs.

### IHC analysis

Four-micrometer-thick, 4%formalin-fixed, paraffin-embedded tissue sections of 27 head and neck tumor tissues, including nasopharyngeal carcinoma (n = 10), tongue carcinoma (n = 7), laryngeal carcinoma (n = 7), other forms of head and neck carcinoma (n = 3), and normal tissues (n = 15), were subjected to the previously mentioned techniques for dewaxing and rehydration [43]. Antigen retrieval was performed in a microwave oven for 8 min with EDTA Antigen Retrieval Solution (50X) (Beyotime, P0085) at pH 9.0. The slides were immersed in a solution of 3% hydrogen peroxide, incubated in the dark for 25 min at ambient temperature, submerged in phosphate-buffered saline (pH 7.4), and rinsed thrice with shaking on a destaining shaker (5 min per wash). Subsequently, the slides were subjected to overnight incubation at 4° C with primary rabbit polyclonal antibodies against 1:200, 1:1000, and 1:200 dilutions of DKK1 (Proteintech; 21112-1-AP), CD3 (Proteintech; 17617-1-AP), or CD4 (Abeam; ab133616), respectively. Next, we used the 2-Sstep Plus® Polyhorseradish Peroxidase (HRP) Anti-goat/Rabbit Immunoglobulin G [IgG] Detection System (Abcam, Ab6721), as per the recommendations provided by the manufacturer. Color development and comparisons were performed using a DAB Kit (Solarbio; DA1010) and hematoxylin, respectively.

All the stained slides were independently scored by two researchers. IHC staining values of 0 (negative), 1+ (weak), 2+ (moderate), and 3+ (strong) for cancer cells were used for semiquantitative assessments in each case. The total score, ranging from 0–300, was derived by computing the percentage of positively stained tissues (1 × x% + 2 × x% + 3 × x% = total score) [44].

### UCSC Xena database

UCSC Xena (http://xena.ucsc.edu/) is a database devoted to genomic data [45]. It enables the analysis of CNVs, somatic mutations, gene expression levels, and methylation. Moreover, the database provides clinical data on patient survival and therapy.

### cBioportal

A collection of cancer genomics datasets is available on the cBioPortal for Cancer Genomics (http://www.cbioportal.org) [46]. DKK1 CNAs and mutations were examined in HNSCC and other malignancies.

### Statistical analysis

DKK1 expression levels were examined using the TIMER, UALCAN, and Oncomine databases. The KM plotter database, along with the R project “survival” and “ggplot2” tools were used to annotate survival curves and forest maps. To assess the link between DKK1 gene expression and the TIMER database, we conducted Spearman’s correlation analysis. For statistical analysis, immunohistochemical data were imported into GraphPad Prism (version 8.0). The means of two or more groups were compared using t-tests and one-way analyses of variance. The “pROC” package (for analysis) and “ggplot2” package (for visualization) were used to analyze ROC curves. P < 0.05 denoted the significant level.

### Data availability statement

All data generated or analyzed during this study are included in this article. Additionally, the raw data supporting the findings of this study are available from the corresponding author upon reasonable request.

### Publication consent

Everyone’s data included in the study was approved for publication.
